# Sensory Attenuation of Self-Produced Feedback: The Lombard Effect Revisited

**DOI:** 10.1371/journal.pone.0049370

**Published:** 2012-11-08

**Authors:** Amanda S. Therrien, James Lyons, Ramesh Balasubramaniam

**Affiliations:** Sensorimotor Neuroscience Laboratory, Department of Kinesiology, McMaster University, Hamilton, Ontario, Canada; The University of Western Ontario, Canada

## Abstract

The Lombard effect describes the automatic and involuntary increase in vocal intensity that speakers exhibit in a noisy environment. Previous studies of the Lombard effect have typically focused on the relationship between speaking and hearing. Automatic and involuntary increases in motor output have also been noted in studies of finger force production, an effect attributed to mechanisms of sensory attenuation. The present study tested the hypothesis that sensory attenuation mechanisms also underlie expression of the Lombard effect. Participants vocalized phonemes in time with a metronome, while auditory and visual feedback of their performance were manipulated or removed during the course of the trial. We demonstrate that providing a visual reference to calibrate somatosensory-based judgments of current vocal intensity resulted in reduced expression of the Lombard effect. Our results suggest that sensory attenuation effects typically seen in fingertip force production play an important role in the control of speech volume.

## Introduction

It is commonly observed that when trying to speak with someone who is listening to music over headphones, they will respond loudly and sometimes even shout. This automatic and involuntary increase in vocal intensity that speakers exhibit in a noisy environment is known as the Lombard effect, named after French otolaryngologist, Étienne Lombard [1]–[9]. Despite the large body of literature that has been published since its initial discovery in 1911, the precise mechanism behind the Lombard effect remains unclear.

Lombard (1911) initially attributed his observations to an automatic self-monitoring process involving auditory feedback [4]. As a result, studies of the Lombard effect have typically focused on the relationship between vocal output and auditory input. Important to remember, however, is that somatosensory feedback from the articulators is equally important for accurate vocal control. Indeed, this has been corroborated by findings in the speech motor learning literature. Alterations of auditory feedback have been shown to induce compensatory changes to pronunciation, demonstrating that the central nervous system actively monitors somatosensory errors signals [10]. In addition, both normally hearing and post-lingually deaf adults have shown adaptation to perturbing loads applied to the jaw during speech, despite those loads producing no measurable acoustical change [11]–[13].

With respect to the Lombard effect, there exists evidence showing that individuals can be trained to use non-auditory sources of sensory feedback in the regulation of vocal intensity. Tonkinson (1994) found that experienced singers were able to learn to use instructions to consciously resist the Lombard effect when performing in chorus [8]. Pick et al. (1989) also examined the effect of instructions on individuals' ability to inhibit the Lombard effect in an unconstrained, free speech task [6]. When simply instructed to resist any changes in vocal intensity, participants were unsuccessful; however, when visual feedback of their vocal intensity was provided, participants could be trained to inhibit the Lombard effect in conditions where masking noise prevented any auditory feedback of their vocal output. The authors suggested that visual feedback of vocal intensity might serve to calibrate somatosensory information from the speech effectors, which allowed participants to use this feedback to maintain a steady voice level. Inherent in this interpretation is the assertion that somatosensory feedback, on it own, is unreliable in generating perceptions of motor output.

Changes in vocal intensity are mediated through changes in subglottic pressure, which are achieved through adjustments of expiratory force [7]. Previous work from our laboratory studying self-produced, discrete, repetitive finger forces has noted automatic and involuntary increases in output when visual feedback of force level is removed. [14]–[16]. These increases in force output were attributed to sensory attenuation mechanisms affecting perceptions of self-produced somatosensory feedback. Specifically, it has been proposed that corollary discharge from primary motor cortex is used to generate predictions of the sensory outcomes of an action [17]–[22]. When that action is executed, the predicted outcomes are compared with incoming afferent information in order to evaluate the success of motor execution as well as to discern self-produced from externally generated feedback [17]–[22]. The comparison process is thought to result in attenuation of the predicted component of incoming sensory information and this attenuation may be responsible for a reduced percept of self-generated sensory feedback compared with that from external sources [17]–[21]. In the case of force production, self-produced forces are perceived as being weaker; therefore, in the absence of more reliable reference stimuli, participants exhibit a compensatory over-production of the force magnitudes required.

It is important to underscore that re-afference mechanisms do not operate exclusively in situations of tactile perception and force production. Indeed, the attenuation of self-generated changes in visual feedback is thought to aid in maintaining stability of the visual scene during eye movements [23]. These attenuation processes, however, render sensory signals from self-movement less reliable. When other feedback modalities are present, they are used to calibrate attenuated somatosensory-based judgments of performance and modulate motor output in subsequent actions. Situations similar to the Lombard phenomenon involve auditory information being rendered unreliable due to increases in levels of ambient noise. As a result, to estimate vocal intensity, the Central Nervous System (CNS) must shift its reliance to favor somatosensory feedback from the speech effectors. Due to the abovementioned sensory re-afference mechanisms, perceptions of self-generated somatosensory feedback are attenuated. In vocal control, reduced salience of somatosensory information could lead to a compensatory increase in vocal intensity following removal of auditory reference stimuli.

If sensory attenuation of somatosensory feedback also underlies the increases in vocal intensity associated with the Lombard effect, then provision of another form of sensory reference, such as visual feedback of vocal output, should calibrate attenuated somatosensory signals and result in reduced positive errors in vocal intensity level following removal of auditory feedback. The objective of the present study was to examine the interplay between auditory and somatosensory feedback modalities in the control of vocal intensity by having participants perform a repetitive vocalization task while auditory and visual feedback stimuli were independently manipulated. We hypothesized that providing a visual reference of participants' voice level would serve to calibrate somatosensory-based judgments of current vocal intensity. We contend that this would result in reduced expression of the Lombard effect when auditory feedback was masked, compared with conditions where no reference stimuli were provided.

## Methods

### Ethics Statement

The study was conducted in accordance with the Declaration of Helsinki and the protocol was approved by the McMaster University Research Ethics Board. Written informed consent was obtained from all participants prior to their participation in the study.

### Participants

Eight participants volunteered for this experiment (6 male and 2 female, mean age: 22.0 years). All participants were students at McMaster University, free of any known speech or hearing impairments and had normal or corrected-to-normal vision at time of data collection.

### Apparatus

Participants spoke in to a small, collar-mounted microphone (Nexxtech omni-directional PC microphone) that was placed out of the breath stream and at a fixed distance of approximately 8 cm from the lips. The microphone output was fed directly to the microphone input of a PC workstation (Dell Precision T7500) where it was recorded by the on-board sound chip set (Intel SoundMAX), then sampled at a rate of 44 100 Hz and processed using custom-written LabView software (LabView 8.5, National Instruments). This software fed the speech signal to a visual feedback display on a 24 inch LCD computer monitor as well as to the computer headphone output. Participants received all auditory feedback through a pair of noise-attenuating headphones (Sennheiser HD280 Pro) connected directly to the headphone output of the PC workstation. In experimental conditions, LabView software delivered a 90 dB pink noise signal to both earphones in place of the microphone output.

### Procedure

Participants sat in a non-moving chair with their arms resting comfortably in their lap. They were fitted with a pair of headphones, a small, collar-mounted microphone and were positioned so they could comfortably see the visual display while maintaining a seated upright posture. Participants were reminded to keep this posture throughout the experiment in order to maintain a constant distance between the microphone and lips. During the experiment, participants were asked to repeat the phoneme, /ba/, at an utterance rate specified by a visual metronome, in the form of a blinking light on the visual display, that was set at 1 Hz (corresponding to 1000 ms between blinks). Participants were to time each utterance with the blink of the metronome. Participants were also presented with a visually specified target volume level of 80 dB SPL and were asked to match it by modulating the intensity of their voice with each successive utterance of the phoneme, /ba/. The volume target was presented as a red line on a continuous line graph on the computer monitor. A second yellow tracing provided online feedback of participants' vocal intensity. The system gain was set so that a 1 dB SPL increase in vocal intensity caused a 1 cm increase in the amplitude of the trace. All trials lasted 20 seconds.

**Figure 1:**
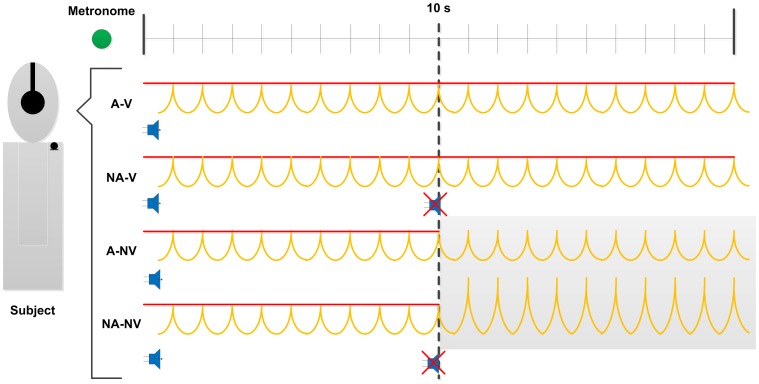
Schematic illustrating the four experimental conditions used in our protocol. Auditory voice feedback either remained present throughout the trial (A), or was replaced with masking noise after 10 s (NA). Similarly, visual feedback of vocal intensity relative to the 80 dB SPL target either remained present throughout the trial (V), or was removed after 10 s (NV). Following feedback manipulations, participants were instructed to make continued responses synchronized with the visual metronome and at the target vocal intensity for the remainder of the trial. We hypothesized that provision of visual reference stimuli would calibrate attenuated somatosensory signals and result in reduced expression of the Lombard effect following removal of auditory voice feedback.

An illustrated depiction of our experimental conditions can be viewed in Figure 1. During the experiment, the visual display of vocal intensity and the auditory voice feedback were manipulated independently resulting in four experimental conditions: the visual feedback of vocal intensity being removed 10 s in to the trial (A-NV), the auditory voice feedback being replaced with masking pink noise 10 s in to the trial (NA-V) or both occurring 10 seconds in to the trial (NA-NV). Following the feedback manipulation, participants were required to continue uttering the phoneme, /ba/, in time with the visual metronome and at the same vocal intensity level for the remainder of the trial. In control conditions (A-V) both visual feedback of vocal intensity and auditory voice feedback remained present throughout the trial. Participants were not informed of the experimental condition prior to beginning each trial. They were given up to 5 practice trials with each condition prior to data collection in order to familiarize themselves with the task and experimental apparatus. During data collection all conditions were presented in a pseudo-randomized order, with each condition being repeated twice before beginning the sequence over. 10 repetitions of each condition were performed, yielding a total of 40 trials per participant.

### Data Analysis

Pressure data (Pascals, Pa) from the microphone output were stored separately for offline analysis. To avoid contamination from transient changes in behavior as participants adjusted to each new trial, the first 2 seconds of data were discarded. To avoid contamination from synchronization errors associated with the visual metronome, the last 2 seconds of data from each trial were also discarded. A custom script in MATLAB^TM^ was used to convert pressure values from Pa to dB SPL, as well as extract the peak dB SPL produced with each utterance, the corresponding sample number and time at which they occurred in the trial. From these data, trials means were computed. Overall vocal intensity was determined as the mean peak dB level from each utterance produced in the last 8 seconds of each trial. Variability was quantified using measures of standard deviation (SD) and coefficients of variation (CV). The vocal intensity-time series produced with each trial were broken down in to mean vocal intensities for two trial phases: before feedback removal (i.e. t = 2–10 s) and after feedback removal (i.e. t = 11–18 s). The difference between these means was then calculated to determine the change in mean vocal intensity over the two trial phases. Lastly, the mean onset time of the 10^th^ utterance (corresponding to t = 10 above) was calculated as this utterance corresponded to the time point when feedback manipulations occurred. Any asynchrony with the metronome on this utterance would have affected the trial phase (described above) in which it occurred. Means were calculated across 15 repetitions of each condition as well as across participants.

### Statistical Analysis

SPSS software (SPSS 16.0) was used to conduct separate analysis of variance (ANOVA) with repeated measures for each dependent variable. Overall vocal intensity, change in mean vocal intensity, vocal intensity variability as well as the iteration time for the 10^th^ utterance were assessed using factors of auditory feedback condition (A, NA) and visual feedback condition (V, NV). Post-hoc means comparisons were performed using Tukey's HSD.

## Results

**Figure 2:**
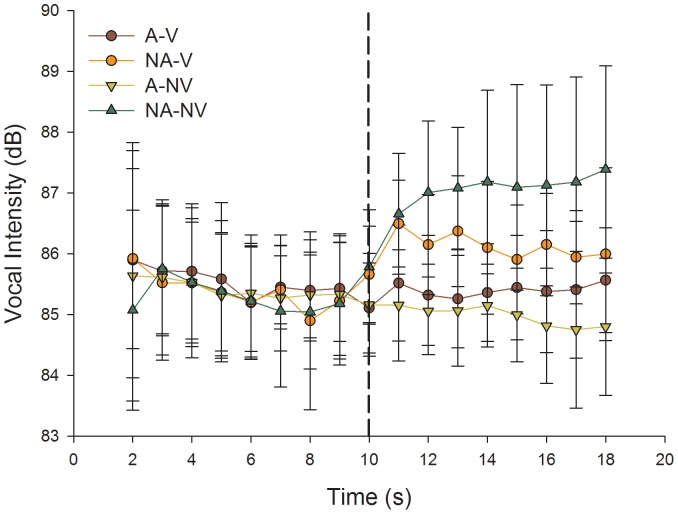
The vocal intensity time series for all conditions, grand averaged across participants. Error bars represent SD. The vertical dashed line represents the time at which auditory voice feedback, visual feedback of vocal intensity or both were removed.

**Figure 3:**
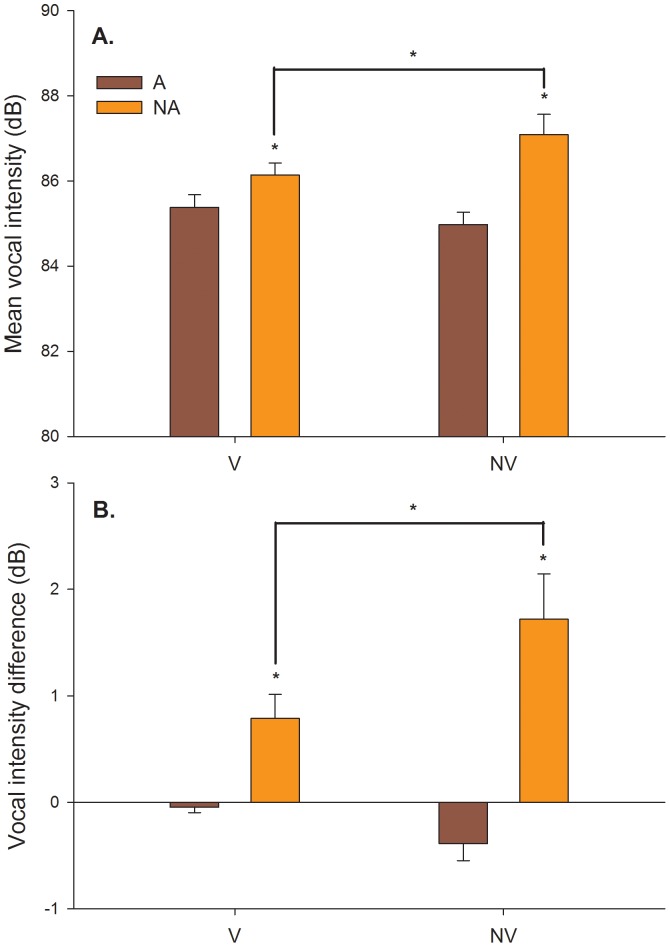
Significant interactions for overall vocal intensity (A) and for the difference in mean vocal intensity over the two trial phases corresponding to the period before feedback removal (t = 2–10 s) and the period following it (t = 11–18 s) (B) among factors of auditory feedback condition and visual feedback condition. In all cases, asterisks and connecting lines represent reliable pairwise comparisons, significant at *p*<.01. Error bars represent standard error.

The average vocal intensity time series obtained from our data can be seen in **Figure 2**. Analysis of overall vocal intensity yielded a significant interaction among factors of auditory feedback condition and visual feedback condition (*F* (1,7) = 13.759, *p*<.01, η^2^ = .663, **Fig. 3A**). Post-hoc means comparisons revealed overall vocal intensity to be significantly greater in the absence of auditory voice feedback regardless of visual feedback condition; however, overall vocal intensity was greatest when both auditory and visual feedback were absent (*p*'s<.01). The ANOVA for change in mean vocal intensity also yielded a significant interaction among factors of auditory feedback condition and visual feedback condition (*F* (1,7) = 10.478, *p*<.01, η^2^ = .599, **Fig. 3B**). Post-hoc means comparisons revealed that, again, the mean change vocal intensity was greater and more positive in the absence of auditory feedback, regardless of visual feedback condition; however, the change was greatest and most positive in the absence of both auditory and visual feedback (*p*'s<.01). Examination of Figure 2 reveals a trend for vocal intensity on the 10^th^ utterance (corresponding to the utterance at which feedback was removed) to be slightly, though not significantly, greater in the two NA conditions, compared to the same utterance in trials where auditory feedback remained present throughout the trial. Analysis of the 10^th^ utterance in all conditions revealed mean response times of 10. 265±0.055 s (A-V), 10.233±0.097 s (NA-V), 10.234±0.132 s (A-NV), and 10.233±0.052 s (NA-NV). Together these yielded an average response lag of 0.241±0.016 s for the 10^th^ utterance. ANOVA for the 10^th^ utterance means revealed no significant main effects or interactions (*p's*>.05), suggesting no significant differences between experimental conditions. Further inspection of Figure 2 revealed great variability in the vocal intensities produced across participants, a finding that is commonly noted in the auditory perturbation literature. Grand mean variability, collapsed over all four experimental conditions was 1.172±0.068 dB and 0.014±0.001 dB for SD and CV values respectively. Despite the large between-subject variability, analysis of SD and CV values did not yield any significant main effects or interactions (*p's*>.05); thus, variability of vocal output intensity was not differentially affected by the removal of auditory and/or visual feedback in our task.

## Discussion

The objective of the present study was to investigate the effects of auditory and visual feedback manipulations on expression of the Lombard effect in a non-communicative, repetitive vocalization task. Previous work from our lab has found that following removal of visual feedback, self-produced, repetitive, discrete finger forces were greater than target magnitudes produced when visual feedback of force output was provided [14]–[16]. These results were interpreted in the context of sensory attenuation mechanisms, whereby self- generated forces were perceived as being weaker leading to a systematic overproduction of the force magnitudes required. Changes in vocal intensity associated with the Lombard effect show a similar pattern of automatic and involuntary increases when the, more reliable, auditory feedback of one's own voice is masked by background noise. If sensory attenuation mechanisms also underlie these increases in vocal intensity, we expected that providing a visual reference of vocal output would result in decreased expression of the Lombard effect in conditions where auditory feedback was masked.

In accordance with previous literature, vocal intensity levels in the present experiment immediately increased when auditory voice feedback was masked with noise, regardless of visual feedback condition. This overall effect of auditory feedback on expression of the Lombard effect is in line with previous research suggesting a central reliance on audition [2], [24]. The degree of increase in vocal intensity was reduced, however, when visual feedback of output volume was provided in combination with auditory masking noise. This result is in line with those of Pick et al. (1989) and suggests a special role for somatosensory feedback from self-produced speech in expression of the Lombard effect [6]. Measures of variability did not reveal any significant main effects or interactions, suggesting that variability associated with vocal output was not differentially affected by the auditory or visual feedback manipulations employed in this experiment. There was a non-significant trend in both NA conditions for vocal intensity on the 10^th^ utterance, corresponding to the utterance where feedback was removed, to be slightly greater compared with conditions where auditory voice feedback remained present throughout the trial. Statistical analysis of response times at this utterance revealed that average responses lagged slightly behind the metronome – a result that is in line with previous work studying asynchronies associated with the use of a visual metronome [25]. Removal of both visual and auditory feedback stimuli was synchronized with the metronome; therefore, both were removed simultaneously with the 10^th^ metronome blink. Considering this, the trend for a slight increase in vocal intensity is likely an artifact resulting from peak intensity levels that were obtained from responses lagging behind the metronome, when both visual and auditory voice feedback had already been removed. The notion of vocal responses to auditory feedback perturbations occurring on such time scales is in line with previous studies of vocal adaptations to pitch-shifted feedback [26], [27]. Overall, these results indicate that the central nervous system is not normally prepared to use somatosensory information from the speech effectors as a primary source of sensory feedback when trying to control vocal intensity. However, when participants are provided with an alternate source of feedback to calibrate somatosensatory information, they are able to regulate their vocal intensity and resist the Lombard effect.

When Lombard (1911) first discovered his effect, he attributed it to processes of internal self-monitoring that required auditory feedback of the voice in order to maintain consistent vocal intensity [4]. Subsequent attempts to elucidate the Lombard effect's underlying mechanism have shown a primary focus on the relationship between vocal output and auditory input. Given the results of the present experiment, as well as work studying repetitive production of discrete finger forces [14]–[16], we propose an alternate mechanism that is centered on the processing of somatosensory feedback from self-produced vocalizations.

Specifically, we propose that increases in vocal intensity associated with the Lombard effect may, at least in part, be the result of compensation for the sensory attenuation of self-produced somatosensory feedback. Vocal production involves motor commands being sent from primary motor cortex to the articulators. Corollary discharge from the motor cortex is used in the generation of predictions of the sensory consequences those commands will yield [17]–[22]. When vocal action is executed, the predicted sensory outcomes are compared with incoming afferent signals in order to evaluate the success of motor execution and distinguish self-produced from unexpected sensory feedback [17]–[22]. Many have hypothesized that this comparison process results in attenuation of the predicted component of incoming sensory signals resulting in a reduced percept of self-generated sensory feedback compared with that which was unexpected or externally-sourced [17]–[21]. The effects of this attenuation process have been shown previously in cases of tactile sensation and peripheral force production [14]–[21]. In the case of vocal control and the Lombard effect, self-produced vocalizations are perceived as being of lower intensity; therefore, without more reliable sensory information to calibrate vocal output, participants automatically and unconsciously increase vocal intensity to compensate.

The notion of such a mechanism in the control of vocalization is well supported by current literature. Neurophysiological evidence of sensory attenuation during self-produced vocalizations has been found in the form of reduced auditory cortex activity in both humans [28]–[31] and non-human primates [32]–[34]. Somatosensory association cortex in humans has also been found to show suppressed activation during self-produced speech relative to silent repetitive movements of the tongue and jaw [35]. Finally, Paus et al. (1996) showed that speech-related motor activity modulated changes in cerebral blood flow to secondary auditory cortex, demonstrating the existence of direct motor-to-sensory feedback regulation in vocal control centers of the human brain [36].

With respect to the Lombard effect itself, indirect evidence for the presence of internal models based on sensory predictions can be drawn from previous work showing that individuals can be trained to inhibit increases in vocal intensity over the long term [6], [8]. Furthermore, the Lombard effect has been shown in a wide variety of non-human animals ranging from primates to whales, which suggests a more generalized mechanism than one specific to humans [7]. A recent study by Love and Bee (2011) failed to show the Lombard effect in tree frogs, leading the authors to suggest that the phenomenon could not be generalized to all vertebrates [37]. The mammals in which the Lombard effect has been demonstrated possess a higher evolved cerebellum relative to reptiles [38]. The cerebellum has been proposed as a likely neural locus for the formation and evaluation of sensory predictions, which are processes integral to mechanisms of sensory attenuation [19].

Many studies investigating the control of vocal intensity have noted a reversal of the Lombard effect, known as the Sidetone effect, in situations where enhanced auditory feedback of the voice is provided in place of masking noise [1], [39]–[40]. Indeed, the results of the present study showed a non-significant trend for overall vocal intensity to decrease in trials where only auditory voice feedback was provided. Computational frameworks of motor control offer a parsimonious explanation of these results [2]. Auditory feedback delivered at a volume levels greater than the vocal intensity of the speaker would be discrepant with central predictions of sensory feedback. To reduce this discrepancy, subsequent motor commands would then be updated to produce a lower vocal intensity on the next utterance. More direct study of the Sidetone effect is needed, however, before specific mechanisms can be implicated in its expression.

In this article, we presented results examining the Lombard effect in a non-communicative, repetitive vocalization task. Our results show that both auditory voice feedback and somatosensory information from the speech effectors are important in the regulation of vocal intensity. We propose a possible for mechanism for the Lombard effect that centers on mechanisms of sensory attenuation affecting somatosensory feedback from self-produced vocalizations. While this mechanism is currently speculative, its role in the expression of the Lombard effect warrants further study. Changing the relative weighting of various sensory feedback modalities in response to auditory feedback perturbations like those seen in the Lombard effect, alters the speech effector system as a whole. Erickson (2002) found that increases in vocal intensity on emphasized syllables could be accomplished through movements of the jaw and tongue [41]. It would be of interest, then, to study the behavior of the supraglottal articulators to determine whether similar compensatory strategies are employed in expression of the Lombard effect.

Aside from increases in vocal intensity, the Lombard effect has been associated with automatic and involuntary changes to other vocal parameters, such as pitch [1]–[9]. In addition, many studies have found enhanced expression of the Lombard effect in communicative situations [1]–[3], [5], [42]. The mechanisms controlling voice pitch are complex and pitch-shifted auditory feedback has been shown to induce other automatic, involuntary changes to vocal output [7], [43]–[44]. Recent evidence suggests that perturbations to vocal pitch and intensity may be processed differently in the auditory cortex of non-human primates [34]; therefore, it is possible that vocal modulations of pitch and intensity in the Lombard effect may be controlled independently. Nonetheless, more study is needed to elucidate the relationship between somatosensory and auditory feedback modalities in the regulation of vocal parameters other than output intensity, especially in situations where verbal comprehension is stressed.
